# A Case of Airway Compromise in a 15-year-old Girl With Intellectual Disability

**DOI:** 10.7759/cureus.14824

**Published:** 2021-05-03

**Authors:** Courtney Haviland, Brian M Cummings, Josephine Lok, Sarah Murphy, Phoebe Yager

**Affiliations:** 1 Pediatrics, Massachusetts General Hospital, Boston, USA; 2 Pediatric Intensive Care Unit, Massachusetts General Hospital, Boston, USA

**Keywords:** pediatric intensive care unit(picu), pediatric emergency department, autism spectrum disorder (asd), pica, esophageal foreign body, intellectual and developmental disabilities, general pediatric surgery, pediatric ent

## Abstract

Foreign body ingestion (FoBI) is an important source of morbidity and mortality in the pediatric population. Patients with intellectual disabilities (ID) are at increased risk of FoBI, likely due to the known association between ID and increased rates of pica. In this report, we present the case of a 15-year-old female patient with autism spectrum disorder (ASD) and ID who presented to the emergency department with fever, drooling, and respiratory failure. She required intubation for airway management. A diagnosis of FoBI was made after striking CT images revealed an entire graphite pencil in her esophagus, causing perforation of the retropharyngeal space. Her recovery course was complicated. Shortly after discharge, the patient was readmitted with repeat FoBI and another significant esophageal injury.

Patients with ID who require surgery due to FoBI are at higher risk of complications and often require prolonged hospitalizations compared to their neurotypical peers. Prevention of FoBI in patients with ID constitutes an important aspect of clinical care and requires efforts toward achieving a balance between patient safety and autonomy.

## Introduction

Foreign body ingestion (FoBI) is highly prevalent in the pediatric population, with an estimated 17,500 emergency department visits per year in the US for bronchial foreign body aspiration alone [1]. Also, there have been reports of its increasing incidence over the past 20 years [2]. FoBI can be difficult to diagnose, and up to 20% of pediatric patients with FoBI are misdiagnosed and treated incorrectly for a period of more than a month [1]. While classically framed as an accidental problem of toddlerhood and early childhood, FoBI also occurs in adolescents, with up to 14% of the incidents reported to be intentional and associated with intellectual disability (ID), including in some patients with autism spectrum disorder (ASD) [2]. One possible explanation is the association between ID and increased rates of pica [3]. Overall, patients with ASD are at risk of increased morbidity and mortality, with a 2-10 fold higher risk of premature death compared to the general population [4]. After drowning, asphyxiation and suffocation are the second and third most common causes of death due to accidental injury in patients with ASD [4]. In this report, we highlight the unique aspects of FoBI in this patient population.

## Case presentation

**Figure 1:**
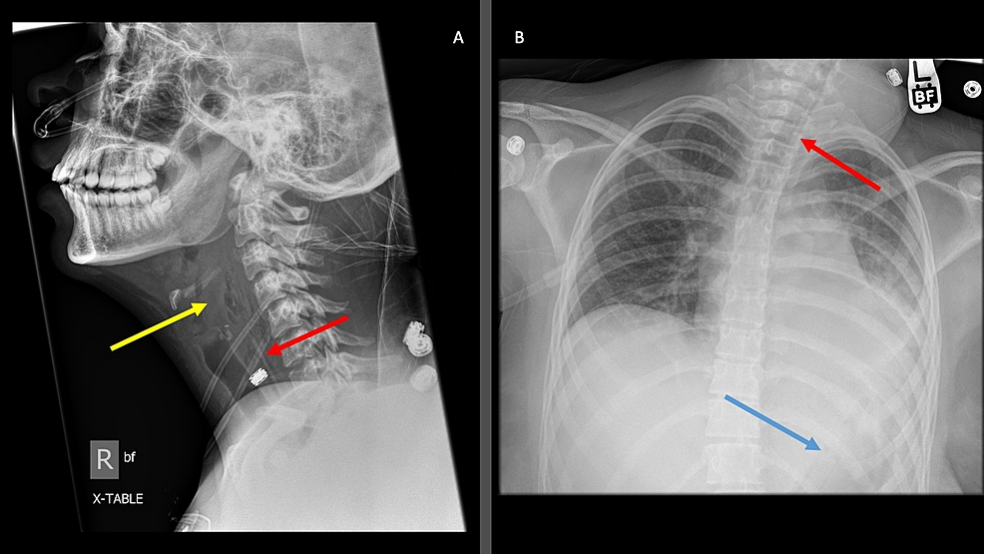
Lateral neck film and chest X-ray A. Lateral neck film showing a thickened epiglottis as well as thickened prevertebral soft tissues with emphysema (yellow arrow). In retrospect, the linear foreign body was noted (red arrow). B. Chest X-ray showing left lower lobe consolidation and effusion and retrospectively noted linear foreign bodies (red arrow and blue arrow)

A 15-year-old, fully vaccinated female with ASD and ID related to perinatal left middle cerebral artery stroke presented to the emergency department (ED) with five days of sore throat and one day of worsening respiratory distress and drooling. She had been previously seen in the ED two days prior for a sore throat, treated with steroids for presumed viral pharyngitis, and subsequently discharged. Upon re-presentation to the ED, she was afebrile, tachycardic to the 120s, with a respiratory rate of 19 breaths per minute. Oxygen saturation was 82% on room air, which improved to 100% after the administration of 100% oxygen by a non-rebreather mask. Chest X-ray showed bilateral infiltrates, and vancomycin and clindamycin were administered for suspected pneumonia. She was transferred to a tertiary care hospital for pediatric intensive care unit (PICU) admission in anticipation of further deterioration. Upon arrival to the PICU, she was awake and alert and sitting in the tripod position with significant drooling. When laid flat for X-ray, she desaturated to 82% on room air. She was administered 10 L of oxygen at 100% FiO_2_ via high-flow nasal cannula, but it still took several minutes for oxygen saturations to recover. A lateral neck film revealed a thickened epiglottis as well as thickened prevertebral soft tissues with emphysema (Figure 1A). Chest X-ray was, at the time, interpreted to show left lower lobe consolidation and effusion (on later review, a foreign body was noted in the area of the esophagus) (Figure 1B). The decision was made to bring her emergently to the operating room (OR) for awake intubation with a surgical airway team on standby. An endotracheal airway was successfully secured.

**Figure 2:**
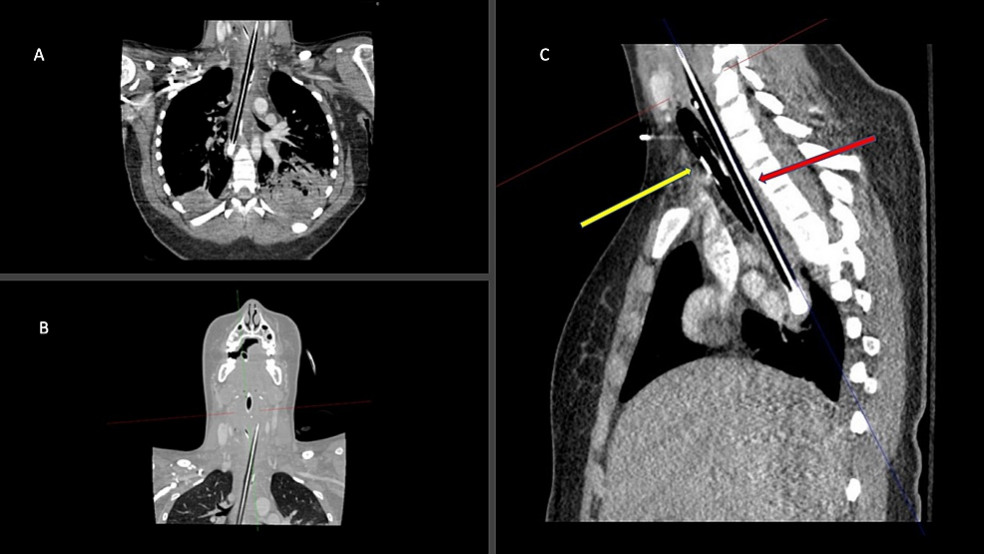
CT images demonstrating graphite pencil (red arrow) lodged in the patient’s esophagus with associated esophageal perforation and retropharyngeal abscess An endotracheal tube (yellow arrow) and nasogastric tube in appropriate position are also visualized CT: computed tomography

**Figure 3:**
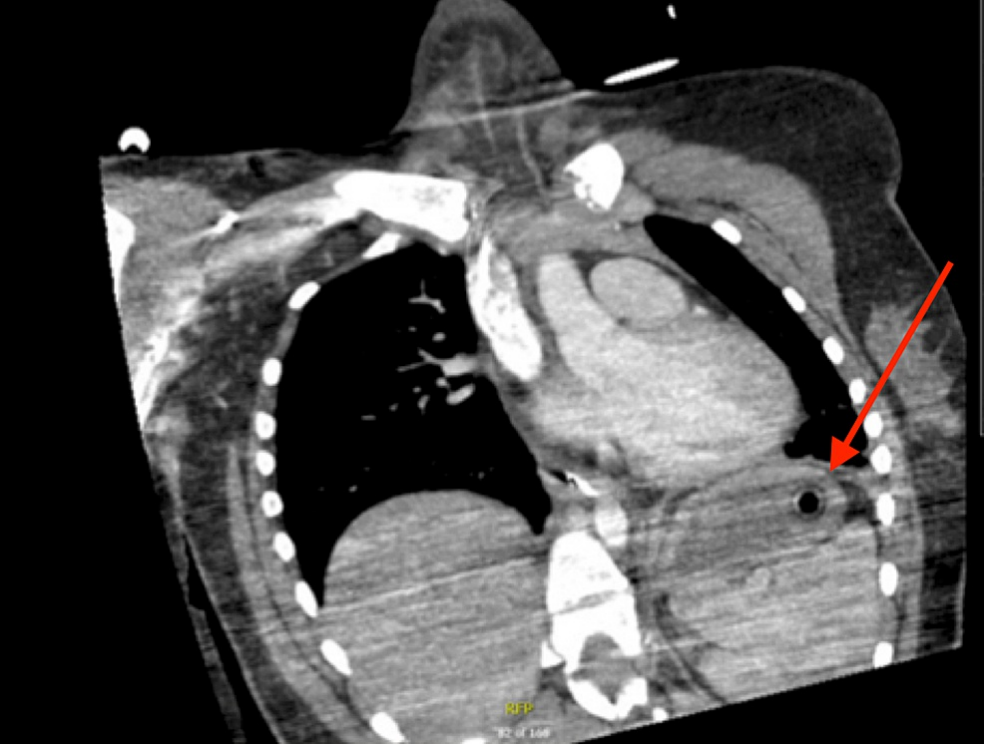
CT image of additional foreign body in the patient’s stomach The foreign body was later retrieved by esophagogastroduodenoscopy and identified as a Crayola marker CT: computed tomography

The differential diagnosis included pneumonia, retropharyngeal abscess, peritonsillar abscess, epiglottitis, caustic ingestion, and FoBI with esophageal or tracheal trauma. CT of the neck and chest was performed, which revealed an approximately 18-cm long cylindrical foreign body in the esophagus with a radio-dense core and peripheral lucency with proximal esophageal perforation and retropharyngeal abscess. The retropharyngeal abscess contained locules of air and extended from the anterior ring of C1 to the C7 vertebral body level (Figure 2). An additional linear object in the stomach was also noted (Figure 3).

The patient underwent esophagogastroduodenoscopy and bronchoscopy with Pediatric Surgery and Otolaryngology. A graphite pencil was removed from the esophagus, and the tip of the pencil was found to have punctured the posterior wall. A retropharyngeal abscess was identified and drained. A Crayola^TM^ marker was also found and removed from the stomach.

The patient was treated with broad-spectrum antibiotics for four weeks, during which she developed drug reaction with eosinophilia and systemic symptoms (DRESS) syndrome to vancomycin, necessitating a steroid course with a five-week taper. In addition, she developed a pulmonary artery embolism, which was thought to be related to medroxyprogesterone for menstrual control, stasis from immobility, and active infection, for which she was treated with heparin. A hypercoagulability panel was found to be normal.

She remained nil per os (NPO) and nasogastric (NG) tube-fed for two weeks, advanced slowly back to a regular diet, and was discharged back to her residential school for continued care after four weeks of hospitalization.

Over the next 18 months, the patient was admitted twice with additional instances of FoBI: the first instance involved a rubber ball that was allowed to pass spontaneously; the second involved a dry erase marker, which resulted in another significant esophageal injury and prolonged hospitalization. During her second hospitalization, a formal report was filed to the Department of Children and Family regarding a concern for child neglect, and soon after discharge, she was transferred to a different residential facility. At the new facility, she continued to require constant monitoring to prevent further ingestions, and new strategies to reduce the risk included a hockey helmet with a face cage as well as intermittent arm restraints when displaying risky behaviors. With these additional safety measures in place, there have been no recurrent injuries from FoBI. Moreover, additional benefits have been seen as these measures have allowed her to engage socially in her group home setting, whereas she had been previously restricted in interactions due to her ingestion risk. At the subsequent follow-up, she was noted to have improved affect, displayed increased interactivity, and was found to be more talkative than prior.

## Discussion

In addition to being at significantly higher risk of FoBI compared to their age-matched peers, patients with ASD and ID constitute a challenging population to diagnose and manage. Contributing factors include difficulty in obtaining a history from non-verbal or non-communicative patients, atypical presentations or localizations of discomfort, and difficulty in performing a thorough physical exam [3,5]. Additionally, about 10% of patients with ASD who are of high-school age and older tend to live in group homes [6], where supervision is distributed across multiple caregivers, potentially leading to delayed recognition of symptoms. When inpatient hospitalization is necessary, patients with ASD and ID often require additional resources, including single rooms and 1:1 sitters, and patients with ID who require surgery as a result of FoBI are at higher risk of complications and often require prolonged hospitalizations compared to their neurotypical peers [3].

This case raised a few ethical and logistical questions for the medical team. Though our patient was found to be pleasant and redirectable, she required a 1:1 sitter throughout her hospitalization to prevent interference with medical devices (NG tube, central venous catheter line, etc.) and further FoBI. After discharge, the patient returned to her residential school and was re-hospitalized a few months later for another esophageal perforation. Apart from the serious threat this posed to the patient’s life, it was also expensive care. The average length of stay for patients with bronchial or esophageal FoBI has been reported to be 11 days, incurring a mean total charge of $34,652 [1]. With two complicated inpatient stays of 27 and 42 days respectively for these potentially preventable injuries, it is likely that the in-hospital cost of this patient highly exceeded the typical charges. Though it could conceivably be safer and more cost-effective for patients such as ours to be kept under constant observation in the community, this is rarely feasible in the current model of residential facility care, which is often understaffed and relies on caretakers who are underpaid, leading to high position turnover [7]. Our patient’s initial management in the community, with reported constant observation and restriction from risky events, had resulted in social isolation and had not been ultimately effective in preventing serious injury. Interestingly, the use of a mechanical barrier (hockey helmet) and intermittent physical restraints allowed our patient more social integration at her residential facility, which led to noted improvements in quality of life and affect, aside from effectively preventing further injury from FoBI.

## Conclusions

FoBI is a significant source of morbidity and mortality in pediatric patients with ASD and ID, and its diagnosis can often be difficult and delayed. Patients with ID are at higher risk of complication and prolonged hospitalization after surgery related to FoBI. Despite the known higher risk in this population, prevention of FoBI remains challenging, with some patients, such as ours, requiring constant supervision and a mechanical barrier to prevent further ingestions. Preventing FoBI in this population requires attention toward achieving a balance between patient safety and autonomy.
